# Fasudil Promotes BMSC Migration via Activating the MAPK Signaling Pathway and Application in a Model of Spinal Cord Injury

**DOI:** 10.1155/2018/9793845

**Published:** 2018-12-30

**Authors:** Jiheng Zhan, Jianbo He, Meihui Chen, Dan Luo, Dingkun Lin

**Affiliations:** ^1^Second College of Clinical Medicine, Guangzhou University of Chinese Medicine, Guangzhou 510405, China; ^2^Laboratory of Osteology and Traumatology of Traditional Chinese Medicine, Lingnan Medical Research Center, Guangzhou University of Chinese Medicine, Guangzhou 510405, China; ^3^Research Laboratory of Spine Degenerative Disease, The Second Affiliated Hospital of Guangzhou University of Chinese Medicine, Guangzhou 510120, China

## Abstract

Bone marrow-derived mesenchymal stem cells (BMSCs) are considered as transplants for the treatment of central nervous system (CNS) trauma, but the therapeutic effect is restricted by their finite mobility and homing capacity. Fasudil (FAS), a potent Rho kinase inhibitor, has been reported to alleviate nerve damage and induce the differentiation of BMSCs into neuron-like cells. However, the effect of FAS on the migration of BMSCs remains largely unknown. The present study revealed that FAS significantly enhanced the migration ability and actin stress fiber formation of BMSCs in vitro with an optimal concentration of 30 *μ*mol/L. Moreover, we found that activation of the MAPK signaling pathway was involved in these FAS-mediated phenomena. In vivo, cells pretreated with FAS showed greater homing capacity from the injection site to the spinal cord injury site. Taken together, the present results indicate that FAS acts as a promoting factor of BMSC migration both in vitro and in vivo, possibly by inducing actin stress fiber formation via the MAPK signaling pathway, suggesting that FAS might possess synergistic effect in stem cell transplantation of CNS trauma.

## 1. Introduction

Spinal cord injury (SCI), a common central nervous system (CNS) trauma, not only causes irreversible neurological dysfunction but also imposes a substantial burden on patients and their surroundings [1]. Despite extensive research and trials of various treatment strategies over the years, the remedy of paralysis after SCI remains elusive [2, 3]. However, the results of infinite proliferation and multilineage differentiation of bone marrow-derived mesenchymal stem cells (BMSCs) have gradually changed the public's pessimistic attitude towards SCI [4]. BMSCs have broad application prospects in the fields of tissue repair and reconstruction [5, 6] and have been used in various clinical trials. Recently, BMSCs have been considered as an alternative strategy for the treatment of SCI because these cells are capable of differentiating into neuron-like cells and participating in axonal regeneration process [7]. However, only a small portion of transplanted cells successfully cross the blood-spinal cord barrier and reach the damaged target tissues or organs, with a relatively low survival rate, thus hindering the widespread application of stem cell transplantation therapy in clinic [8, 9]. Therefore, it is urgently needed to develop an improved approach to enhance BMSC migration and homing for the purpose of better therapeutic effects.

Stem cell-based tissue regeneration requires cells to migrate from the initial sites where they colonize to lesion sites [10]. Actin filament-microtubule interaction, regulated by several distinct but interacting signaling pathways, is needed for whole-cell migration processes [11, 12]. Among them, mitogen-activated protein kinase (MAPK) is the main signaling pathway of cellular motility, proliferation, and apoptosis, which is involved in the maintenance of neuronal hyperexcitability after SCI [13–15]. These findings provide more direction for future clinical treatment.

Fasudil (FAS) is a potent Rho kinase (ROCK) inhibitor with good therapeutic effect in patients with subarachnoid hemorrhage without serious side effects [16, 17]. Expression levels of RhoA and RhoB (human) or ROCK1 and ROCK2 (rat) were upregulated at the injured sites after SCI [18]. By suppressing with FAS, it is possible to block the inhibitory effects of Rho/ROCK pathway during regeneration and normalize the blood flow of the injured sites, thereby further protecting damaged nerve tissues [19]. In addition, Chiba et al. also found that FAS can enhance the axonal regeneration induced by BMSCs transplanted in vivo, thereby exerting neuroprotective effects on SCI [10]. Nonetheless, whether FAS has the potential to promote the migration of BMSCs and whether the possible underlying mechanism occurs via the MAPK signaling pathway remain unclear.

In this study, we evaluated the migration ability of BMSCs treated with FAS in vitro and explored its underlying mechanisms. Additionally, we further investigated the effect of FAS on the homing capacity of BMSCs transplanted into rats subjected to SCI. Our findings provide a theoretical basis for FAS to play a promising role in stem cell transplantation for CNS trauma.

## 2. Materials and Methods

### 2.1. Materials

Specific pathogen-free Sprague Dawley (SD) rats used in this study were provided by the Guangdong Medical Laboratory Animal Center (Foshan, China, certificate no. 44007200047868). All animals were treated according to the animal guidelines of Guangzhou University of Chinese Medicine. *α*-Modified Eagle's medium (*α*-MEM), fetal bovine serum (FBS), and trypsin were obtained from Gibco-BRL (NY, USA). Cell counting kit-8 (CCK-8) was purchased from Dojindo Laboratories (Kumamoto, Japan). FAS was purchased in Aladdin (purity ≥98%; Shanghai, China). SP600125, PD98059, and SB202190 were purchased from Selleck Chemicals (TX, USA). 5′-Bromo-2-deoxyuridine (BrdU) was purchased from Sigma-Aldrich (MO, USA). Transwell system was purchased from Millipore (MA, USA). Rhodamine phalloidin was bought from Yeasen Biotech (Shanghai, China). FAS was dissolved in PBS before dilution with the culture medium.

### 2.2. Cell Isolation and Culture

BMSCs were isolated from the bone marrow of tibiae and femurs of 4-week-old rats using centrifuging and washing as previously described [20]. The cells were seeded into 25 mL culture flasks and cultured in *α*-MEM supplemented with 10% FBS in a standard incubator under a humidified atmosphere of 5% CO_2_ and 95% air at 37°C. The culture medium was carefully changed every 3 days. When most of the cultured cells were identified as BMSCs, cells from 3 to 5 passages were used for the subsequent experiments.

### 2.3. Immunophenotypic Analysis of BMSCs

Upon the third passage, BMSCs were trypsinized and then centrifuged. The cell pellet was then gently resuspended into single cell suspensions in ice-cold PBS containing 0.1% BSA, and the density was adjusted to 3 × 10^6^ cells/mL. Next, about 3 × 10^5^ cells were stained with phycoerythrin- (PE-) labeled primary antibodies against CD44, CD34, and corresponding isotype control antibody to rat IgG1 in the dark at room temperature. After washing twice with ice-cold PBS, the cells were fixed in 1% paraformaldehyde (PFA) and analyzed by flow cytometry to determine the surface marker expression and purity of rat BMSCs.

### 2.4. Differentiation of BMSCs

To determine whether the isolated BMSCs possess multipotent differentiation ability in vitro, BMSCs at passage 4 were cultured in osteogenic induction medium containing 0.01% dexamethasone, 1% *β*-glycerophosphate, and 0.2% ascorbic acid or chondrogenic induction medium containing 0.01% dexamethasone, 0.3% ascorbic acid, 1% insulin-transferrin-selenium+premix, 0.1% sodium pyruvate, 0.1% proline, and 1% TGF-*β*3 for 2 weeks. The induction medium was replaced every 3 days. After differentiation, cells were fixed with 4% PFA for 20 min and subjected to alizarin red staining, alkaline phosphatase staining, and alcian blue staining. The cultures were then washed three times with PBS and photographed using an inverted microscope with a camera.

### 2.5. Cell Proliferation Assay

A CCK-8 assay was performed to evaluate the effect of FAS on the proliferation of BMSCs. BMSCs at passage 4 were seeded in 96-well plates at a density of 1 × 10^4^ cells per well with four replicates for each group. When BMSCs grew to 80% confluence, the cells were serum starved for 12 h and then treated with complete medium with or without different concentrations of FAS (0, 3, 10, 30, and 100 *μ*M) accordingly for 12, 24, 36, 48, and 72 h. At each time point, 10 *μ*L of CCK-8 reagent was added into each well and incubated for further 2 h at 37°C. The optical density was then measured at a wavelength of 450 nm using a microplate reader (Bio-Rad, USA) according to the manufacturer's protocol.

### 2.6. Scratch Wound Healing Assay

BMSCs at passage 3 were seeded in 6-well plates at a density of 1 × 10^6^ cells per well. When cells grew to 90% confluence, the medium was aspirated away, and cells were serum-starved for 12 h. A scratch wound was gently created by using a sterile micropipette tip on a uniform layer of cells, and the cellular debris and floating cells were removed by rinsing with PBS. The cells that migrated from the border of the wound to the wounded area were photographed at 0, 12, and 24 h in the same area after wounding. After fixation, the cells were stained with 0.1% crystal violet (Beyotime, China) for 30 min and observed as described previously [21]. Assays were performed in triplicate.

### 2.7. Transwell Migration Assay

Cell migration was measured using a Transwell system (Millipore, USA) with a polycarbonate filter membrane of 8 *μ*m pore size. The cell density was adjusted to 1 × 10^6^ cells/mL with *α*-MEM containing 1% FBS. The cells were divided into the following groups: (1) control group (0 *μ*M FAS in both the upper and lower chambers); (2) FAS group (upper chamber: 0 *μ*M FAS, lower chamber: 30 *μ*M FAS); (3) FAS + PD group (upper chamber: 0 *μ*M FAS, lower chamber: 30 *μ*M FAS and 20 *μ*M PD98059); (4) FAS + SP group (upper chamber: 0 *μ*M FAS, lower chamber: 30 *μ*M FAS and 20 *μ*M SP600125); and (5) FAS + SB group (upper chamber: 0 *μ*M FAS, lower chamber: 30 *μ*M FAS and 20 *μ*M SB202190). 150 *μ*L of cell suspension was added to the upper chamber, and basal medium containing different reagents was added to the lower chamber. At the end of a 24 h incubation period, all the nonmigrated cells in the upper chamber were removed with a cotton-tipped swab and the cells that migrated through the pores were stained with crystal violet. The cells migrated to the underside of the filters were photographed with optical microscopy, and five visual fields were randomly selected from each insert for counting.

### 2.8. Rhodamine Phalloidin Staining

BMSCs were grown in 24-well plates with one glass coverslip in each well at a density of 300 cells per well. The cells were serum-starved for 12 h and randomly divided into the control group, FAS group, FAS + PD group, FAS + SP group, and FAS + SB group. BMSCs in the control group were treated with *α*-MEM, while those in the experimental groups were reacted with FAS with or without one of the three inhibitors. After treatment, cells were fixed with 4% PFA for 20 min, permeabilized with 0.2% Triton X-100 for 15 min, and blocked with 5% bovine serum albumin (BSA; Biosharp, China) for 30 min at room temperature. BMSCs on coverslips were washed with PBS for three times, stained with 100 nM rhodamine phalloidin for 30 min, then counterstained with 4′,6-diamidino-2-phenylindole (DAPI; Cell Signaling Technology, USA) to label nuclei, and mounted. Cell images were captured with a Leica SPE-II confocal laser scanning microscope (Leica Microsystems, Germany).

### 2.9. Western Blot Analysis

Phospho-extracellular signal-regulated kinase 1/2 (ERK1/2), phospho-c-Jun amino-terminal kinase (JNK), and phospho-p38 protein expression was detected by Western blot. In brief, the cellular proteins from each sample were extracted using a cell lysis buffer. Equal amounts of total protein were separated electrophoretically on 10% SDS-PAGE gel (Beyotime, China) and electrotransferred onto PVDF membranes (Millipore, USA). After blocking with 5% skim milk, the membranes with protein samples were incubated with primary antibodies phospho-ERK1/2 (Abcam, USA), phospho-JNK, and phospho-p38 (Cell Signaling Technology, USA) overnight at 4°C, followed by sequential incubation with horseradish peroxidase-conjugated secondary antibodies for 90 min at room temperature. Images for immunoblot analysis were captured on a chemiluminescence imaging analyzer (Bio-Rad, USA), and the intensities of bands were quantified using Quantity One software (Bio-Rad, USA).

### 2.10. FAS Pretreatment and BrdU Labeling of BMSCs In Vitro

BMSCs at passage 3 were divided into 2 groups (1 × 10^6^ cells in each group): (1) control group and (2) FAS treatment group. FAS was dissolved in PBS to a final concentration of 30 *μ*M. All groups of cells were labeled with 10 *μ*mol/L of BrdU with or without FAS for 3 days. The efficiency of BrdU labeling was observed by a fluorescence microscopy. The cells were then harvested for subsequent experiments.

### 2.11. Contusion SCI Model and BMSC Transplantation

Eighteen adult female SD rats weighing 270 to 300 g were randomly divided into the model group, BMSC group, and FAS + BMSC group. Each animal was subjected to a moderate contusion model of SCI as previously described, with minor modification [22]. A dorsal laminectomy was performed at the T9-T10 levels under sodium pentobarbital (40 mg/kg, i.p.) anesthesia, and then, the spinal cord with dura mater was exposed. Subsequently, a 10 g weight impactor (diameter, 2 mm) dropped from a height of 50 mm onto the exposed dura of T10 level. Penicillin (3 × 10^4^ U/kg) was injected intramuscularly in the first 3 days after SCI. Upon awakening, each rat's bladder was manually pressed twice a day until the autourination function was restored. Seven days after SCI, the rats in the FAS + BMSC group were injected with BMSCs that had been incubated with FAS for 72 h, and the rats in the BMSC group were injected with BMSCs without FAS pretreatment via the tail vein; the model group was injected with the same volume of PBS. The cells were all labeled with BrdU before injection.

### 2.12. Immunofluorescence Assay

Four weeks after surgery, all rats were deeply anesthetized and then perfused transcardially with ice-cold PBS followed by 4% PFA. The spinal cord segments (5 mm centered at the lesion site) were removed, postfixed in the same fixative overnight, dehydrated with 30% sucrose, and embedded in OCT compound. The samples were cut into serial cross sections of 10 *μ*m and mounted onto glass slides. After rinsing three times with PBS, the serial sections were blocked with 5% BSA and incubated overnight at 4°C with a solution containing BrdU primary antibody (Cell Signaling Technology, USA) to determine the distribution of transplanted cells in the spinal cord. Following another three 5 min washes in PBS, sections were reacted with secondary antibody at room temperature for 1 h. The nuclei were counterstained with DAPI for 5 min. Then, the slides were mounted and processed for microscope observation. Positively stained BMSCs were quantified in three random areas of each section.

### 2.13. Statistical Analysis

Data were statistically analyzed and presented as mean ± standard deviation. Differences between two groups were analyzed statistically by unpaired Student's *t*-test. Differences among groups were assessed with one-way analysis of variance (ANOVA). A probability value of *P* < 0.05 was accepted to be statistically significant. All analyses were performed using SPSS 24.0 software.

## 3. Results

### 3.1. Isolation, Culture, and Identification of Rat BMSCs

**Figure 1:**
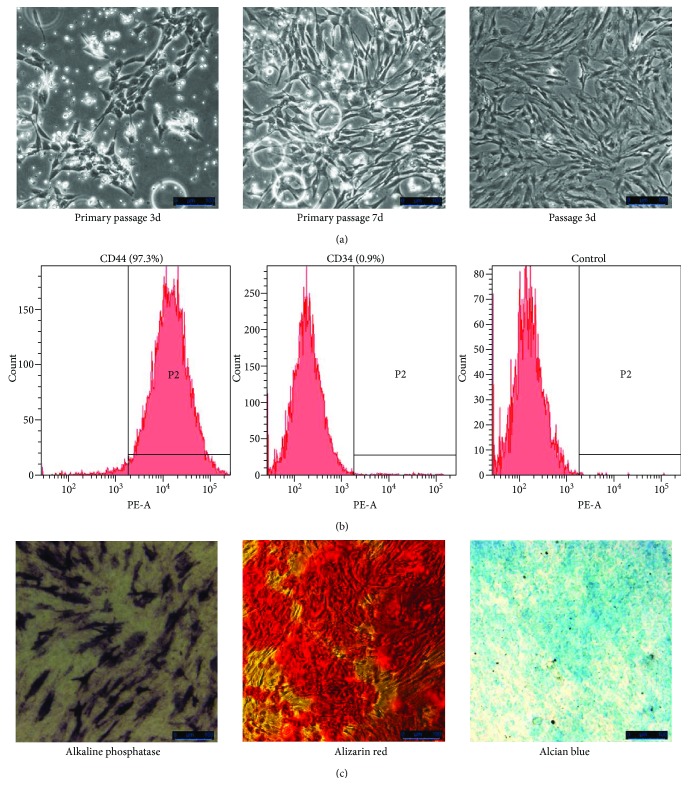
Morphology, phenotypic characterization, and differentiation of rat BMSCs. (a) Representative fields of BMSC morphologies at different passages. (b) The BMSCs were identified with CD44 (97.3%), CD34 (0.9%), and corresponding isotype control. (c) Analysis of osteogenic differentiation using alkaline phosphatase staining and alizarin red staining and analysis of chondrogenic differentiation using alcian blue staining.

At initial phase, rat BMSCs exhibited fibroblast-like shape and spiral arrangement in culture, reaching 80%–90% confluence at day 7 (Figure 1(a)). The floating cells were completely abolished at passage 3 (Figure 1(a)). Flow cytometry data showed that the expression of surface marker and purity of cells were positive to CD44 (97.3%) and negative to CD34 (0.9%), indicating that most of the cells expressed standard surface markers of rat BMSCs (Figure 1(b)). Upon induction, BMSCs consistently differentiated into osteogenic/chondrogenic cells, indicating that the BMSCs were multipotent (Figure 1(c)).

### 3.2. Effect of FAS on the Proliferation of BMSCs

**Figure 2:**
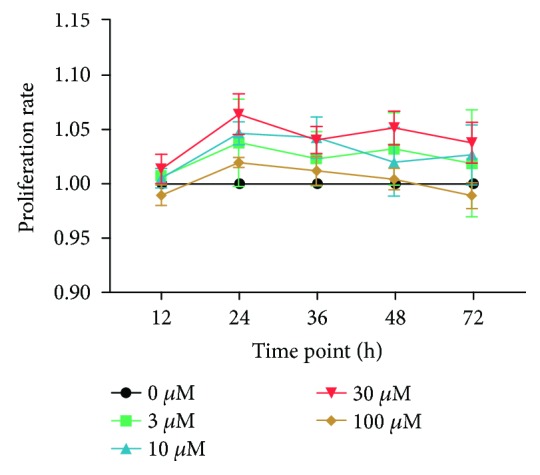
Effect of FAS on the proliferation of BMSCs. BMSCs were treated with various concentrations of FAS (0, 3, 10, 30, and 100 *μ*M) for 12, 24, 36, 48, and 72 h. Line chart showing the proliferation rate of BMSCs as determined by CCK-8 assay.

To study the effect of FAS on BMSC proliferation, cells were cultured in medium and treated with different doses of FAS (0, 3, 10, 30, and 100 *μ*M) for 12–72 h. Then, we measured cell proliferation by the CCK-8 assay. As shown in Figure 2, there were no significant differences between the control group and the FAS treatment groups, indicating that FAS do not noticeably promote BMSC proliferation.

### 3.3. FAS Promotes BMSC Migration In Vitro

**Figure 3:**
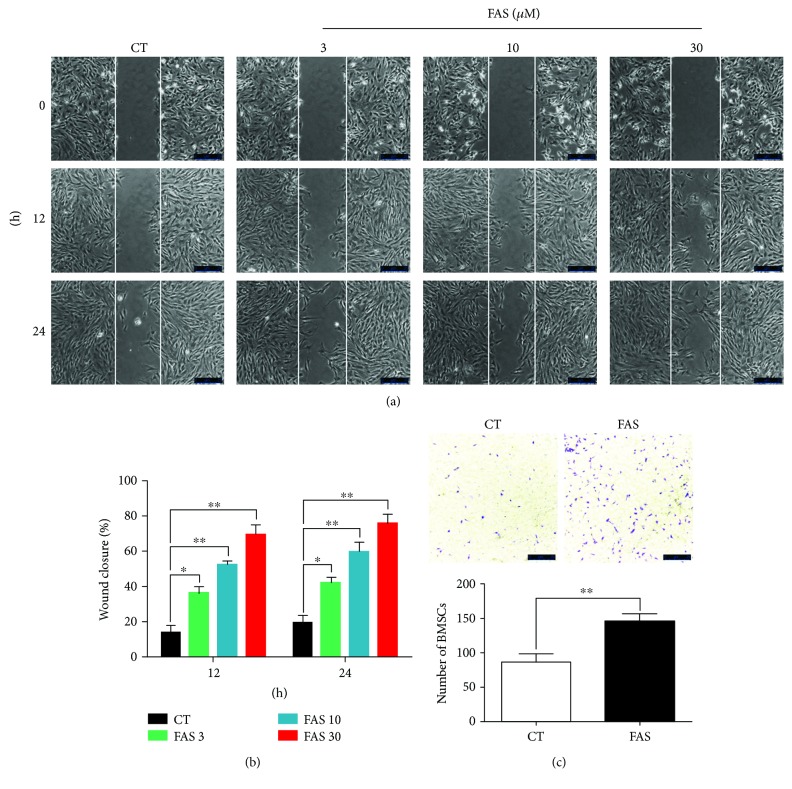
FAS induces BMSC migration in vitro. (a) Scratch wound healing assay of BMSCs treated with 3, 10, and 30 *μ*M FAS. Phase contrast images were captured at 0, 12, and 24 h after the scratch. (b) Data quantified from the experiment shown in (a). Three random fields were selected from each group, and the rate of wound closure was measured using ImageJ software. (c) Analysis of FAS treatment on BMSCs by Transwell migration assay. Phase contrast images of migrated cells taken 24 h later. The result are shown as the mean value of 5 random fields from each insert. The mean and standard deviation of three independent experiments are shown. ^∗^*P* < 0.05 and ^∗∗^*P* < 0.01 versus control (CT) group.

Next, we assessed the effect of FAS on cell migration through the scratch wound healing assay and Transwell migration assay. In the scratch wound healing assay, a large number of cells treated with different concentrations of FAS migrated from the border of the wound to the wound area 24 h after the scratch, while only a small amount of untreated cells were found in the scratch wound (3 *μ*M and 10 *μ*M (*P* < 0.05) and 30 *μ*M (*P* < 0.01); Figure 3(a)). FAS at 3 *μ*M, 10 *μ*M, and 30 *μ*M increased the rate of wound closure of BMSCs to 41.3%, 60.5%, and 72.6%, respectively (Figure 3(b)). Taken together, FAS (30 *μ*M), with potent effects on migration and no significant effects on proliferation, was therefore chosen for the further experiments. Similar results were also obtained in the Transwell migration assay. A greater migration capacity was observed in the FAS-treated groups compared to the control group, and 30 *μ*M FAS leading to a twofold increase in the number of BMSCs that migrated to the lower chamber (Figure 3(c)). Together, these data suggest that FAS promotes BMSC migration with the maximal stimulatory effect at 30 *μ*M.

### 3.4. FAS Enhances BMSC Migration Probably by Stimulating Actin Stress Fiber Formation

**Figure 4:**
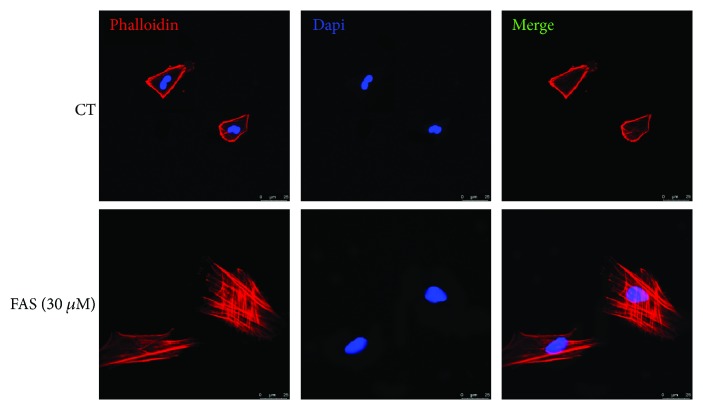
FAS stimulates actin stress fiber formation of BMSCs. BMSCs were serum starved for 12 h and treated with PBS or 30 *μ*M FAS for 4 h. After treatment, rhodamine phalloidin staining was performed to stain actin stress fibers. Nuclei were counterstained with DAPI.

In higher eukaryotes, cells can migrate using different migration modes, while the migrated mesenchymal cells are characterized by the presence of actin stress fibers. To investigate the effect of FAS on actin stress fibers in BMSCs, we detected the distribution and formation of actin cytoskeleton in BMSCs using immunofluorescence. Actin stress fibers were stained with rhodamine phalloidin, and there was a clear deficiency in the ability of actin stress fiber formation in the absence of FAS (30 *μ*M) treatment. This observation was consistent across all cells analyzed (Figure 4).

### 3.5. FAS Upregulates Protein Expression of the MAPK Signaling Pathway

**Figure 5:**
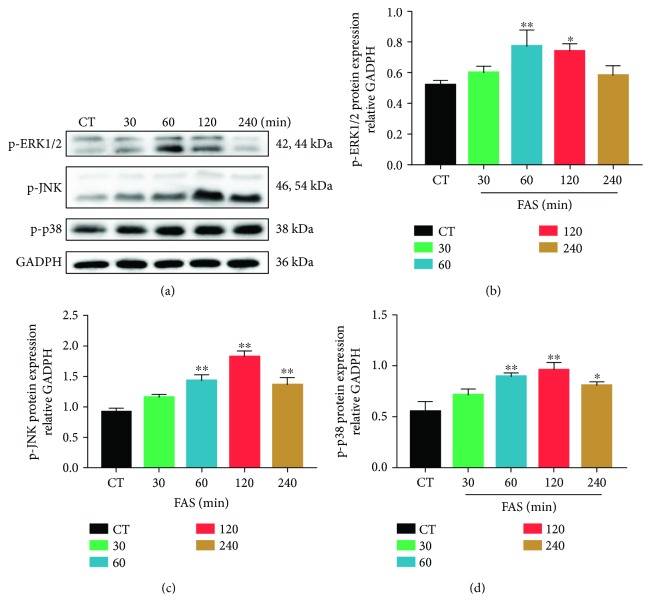
FAS upregulates the protein expression of the MAPK signaling pathway in BMSCs. BMSCs were serum starved for 12 h and treated with 30 *μ*M FAS for 30, 60, 120, or 240 min. Total protein extracts were prepared, and the phosphorylation levels of ERK1/2, JNK, and p38 were detected by Western blot. (a) Representative Western blot images of p-ERK1/2, p-JNK, and p-p38. (b–d) Densitometric analysis of the blots was shown in panel after being normalized by GADPH. One-way ANOVA followed by Tukey's test. Experiments were repeated at least three times. ^∗^*P* < 0.05, ^∗∗^*P* < 0.01 versus control (CT) group.

Activation of the MAPK signaling pathway is critical for cell migration process. To examine whether FAS can upregulate the MAPK signaling pathway, we treated the cells with FAS (30 *μ*M) for 30, 60, 120, or 240 min and measured the expression levels of the phosphorylation status of ERK1/2, JNK, and p38 by Western blot. We found that the expression levels of phospho-ERK1/2, phospho-JNK, and phospho-p38 significantly increased 30 min after FAS treatment and reached the maximum at 60 min, 120 min, and 120 min, respectively (Figure 5).

### 3.6. Activation of the MAPK Signaling Pathway Contributes to FAS-Induced BMSC Migration

**Figure 6:**
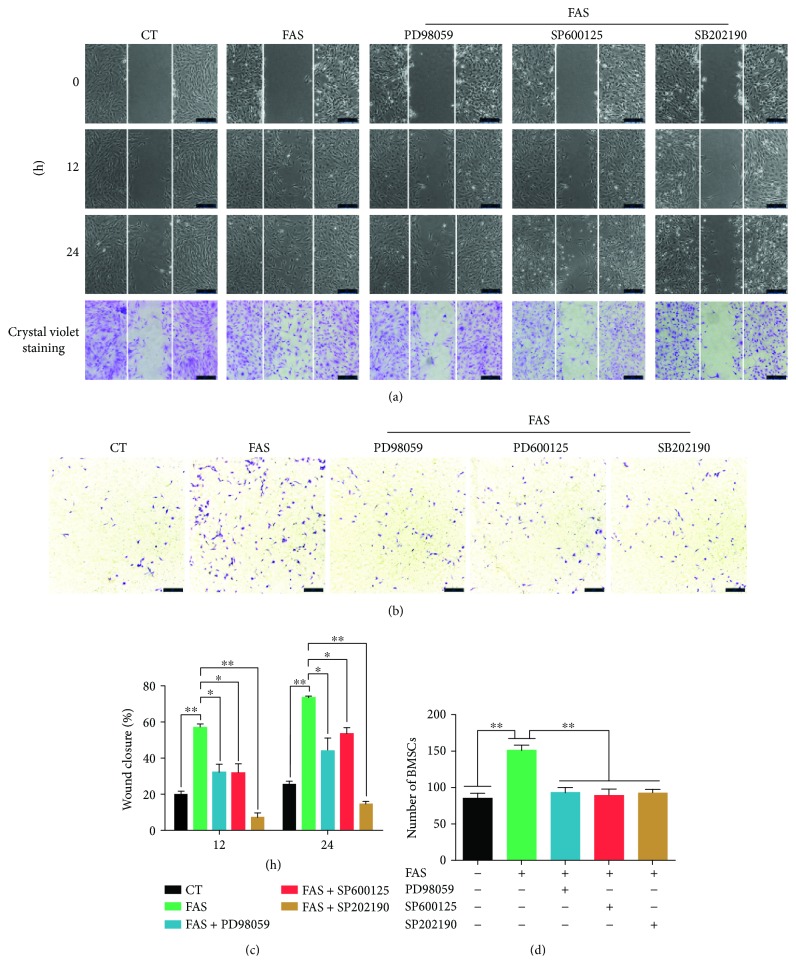
FAS promotes BMSC migration in vitro via activation of the MAPK signaling pathway. BMSCs were treated with FAS in the presence of MAPK inhibitors, and then, we determine the role of the MAPK signaling pathway in FAS-induced migration by using scratch wound healing assay and Transwell migration assay. (a) Phase contrast images were captured at 0, 12, and 24 h after the scratch. (b) Phase contrast images of migrated cells taken 24 h later. (c) Quantified data from the experiment shown in (a). (d) Data quantified from the experiment shown in (b). The means ± standard deviation of three independent experiments are shown. ^∗^*P* < 0.05, ^∗∗^*P* < 0.01 versus FAS group.

**Figure 7:**
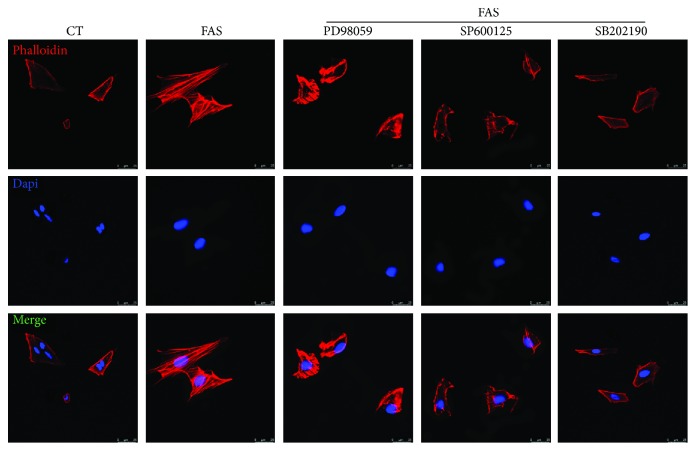
FAS induces actin stress fiber formation by activating the MAPK signaling pathway. BMSCs were serum starved for 12 h, pretreated with one of the three inhibitors for 1 h, and then incubated with FAS. After treatment, rhodamine phalloidin staining was performed to stain actin stress fibers. Nuclei were counterstained with DAPI.

To further study the role of MAPK signaling pathway in the FAS-induced BMSC migration, we analyzed cellular migration ability by the scratch wound healing assay, Transwell migration assay, and rhodamine phalloidin staining in the presence of the specific inhibitor of ERK (PD98059), JNK (SP600125), or p38 (SB202190). As shown in the scratch wound healing assay and Transwell migration assay (Figure 6), the inhibitor groups showed significant reduction of FAS-induced migration of BMSCs. Similar results were also obtained in rhodamine phalloidin staining, and numerous actin stress fibers were arranged across the cell bodies compared to the controls (Figure 7). After treatment with FAS in the presence of the three inhibitors, the quantity of actin stress fibers decreased, and only a small amount of fibers distributed along the cell membrane were observed.

### 3.7. FAS Promotes the Homing of BrdU-Labeled BMSCs in SCI Model

**Figure 8:**
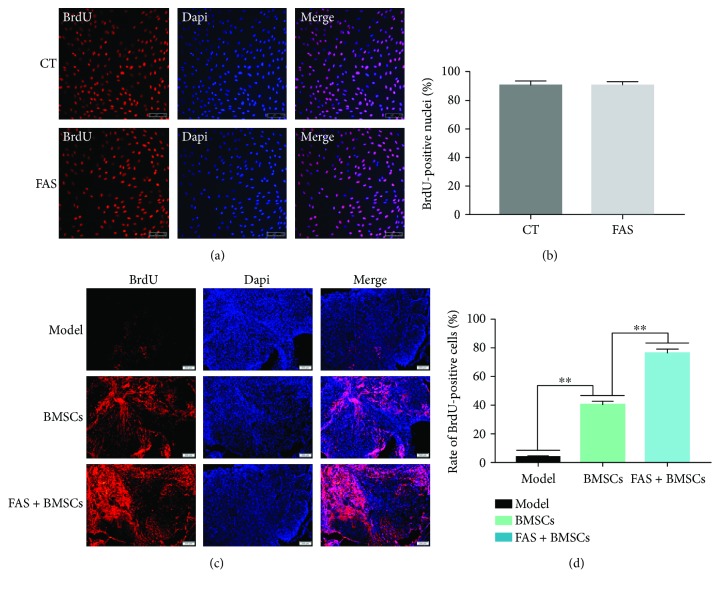
BrdU immunostaining of BMSCs grafted to the spinal cord injury site. (a) Positive cells were labeled with red fluorescence in the nucleus. And BrdU-DAPI staining showed no significant differences in BrdU^+^ BMSCs between the two groups. (b) The labeling efficiency of BrdU in the control (CT) group was 90.6 ± 3.3%, and 90.9 ± 2.4% in the FAS treatment group. (c) The number of BMSCs pretreated with FAS in the spinal cord injured tissue was much greater than that in the group injected with control BMSCs. (d) Statistical data analysis of the positive cell percentage at the spinal cord injury site. ^∗∗^*P* < 0.01 indicates significant differences between groups (one-way ANOVA).

To ensure that most of the BMSCs were labeled with BrdU, the cells were cultured with complete medium containing 10 *μ*M BrdU for 3 days. The result of the immunofluorescence showed that the percentage of BrdU-positive (BrdU^+^) BMSCs in the control group was 90.6 ± 3.3% and that in the FAS treatment group was 90.9 ± 2.4% (*P* > 0.05; Figures 8(a) and 8(b)). 21 days after intravenous transplantation, BMSCs that were grafted to the injured spinal cord were tracked using BrdU immunostaining. The implanted cells were identified as red fluorescent cell assemblies of various sizes in the spinal cord. The percentage of BrdU^+^ cells at the injury site in the model group, BMSC group, and FAS + BMSC group were 4.2 ± 0.4, 40.3 ± 1.9, and 76.3 ± 2.3, respectively (Figure 8(c)). As shown in Figure 8(d), the number of BrdU^+^ cells among rats in FAS + BMSC group was approximately twice that of the rats in BMSC group.

## 4. Discussion

Increasing evidence suggests that mesenchymal stem cells are a promising cell source for tissue engineering, tissue regeneration, and gene therapy applications. Mesenchymal stem cells isolated from the bone marrow can differentiate into neuron-like cells and secrete series of neurotrophic factors [23], thus attracting researchers' attention to the treatment of SCI. The use of BMSCs for cell therapy relies on the capacity of these cells to home and engraft long-term into the appropriate target tissue. However, the defective migration potential of BMSCs has been discussed and considered to hamper their effective use in gene therapy or tissue regeneration [24]. Therefore, it is urgent to develop an improved approach to increase the homing rate of BMSCs. In this study, we hypothesized that FAS might enhance the migration of BMSCs. To determine the optimal concentration of FAS for the induction of BMSC migration, concentrations from 0 *μ*M to 100 *μ*M were tested in the CCK-8 assay. We found that FAS had no positive effect on BMSC proliferation. In combination with the scratch assay, we found that FAS at 30 *μ*M induced more BMSC migration than other concentrations of FAS, so this concentration was used in subsequent experiments. Additionally, the pro-migration effect of FAS on BMSCs was further confirmed in Transwell migration. We further showed that FAS promoted the actin stress fiber formation in BMSCs, which may be responsible for its pro-migration effect on BMSCs.

The cytoskeleton consists of three principal polymer systems: microtubules, microfilaments, and intermediate filaments [25]. The actin microfilament network is essential for maintaining cell shape and function in all cells, most notably in eukaryotic cells [26]. It has a multitude of roles in cellular processes such as cell adhesion, motility, cellular signaling, intracellular trafficking, and cytokinesis. A previous study suggested that rho inhibitors such as FAS not only promoted the migration of mesenchymal stem cells in vitro but also inhibited the formation of actin stress fibers [24]. However, some researchers hold opposite views on the relationship between the formation of actin stress fibers and cell migration, most likely due to differences in the species from which the tested cells were obtained. A characteristic of mesenchymal-migrating cells is the presence of actin stress fibers, which are thought to mediate myosin II-based contractility. Myosin II-based contractility regulates various cellular activities that occur in a spatial and temporal manner to achieve directional cell migration [27]. Upon the treatment of mesenchymal cells with FAS, myosin II remodels actin into thin bundles. This action promotes the accumulation of *α*-actinin into these actin-myosin bundles, resulting in the formation and assembly of actin stress fibers [28]. It is worth noting that there is a close interconnectivity among stress fibers, which is likely to ensure spatiotemporal myosin II-based contractility to achieve directional cell migration [29, 30]. Interestingly, some cells, such as leucocytes, migrate faster without actin stress fibers, so some researchers conclude that stress fiber formation inhibit cell migration [31]. Subsequently, this conclusion was dispelled, partly due to a more accurate understanding of the different migration modes and actin stress fiber differences in migrating and stationary cells.

Accumulating evidence indicates that the MAPK signaling pathway plays a critical role in the process of BMSC migration [25, 32–34]. Activation of MAPKs of the early and late phases may be regulated by different mechanisms and has different functions [35], which remain to be clarified. When activated, MAPKs enhanced myosin light chain kinase (MLCK) activity, leading to increased MLC phosphorylation [36]. The phosphorylation of MLC appears to be required for formation and remolding of actin filaments in the cell body [37]. In our study, the stepwise phosphorylation of three prototypic members of the MAPK family, ERK1/2, JNK, and p38, was upregulated 30 min after FAS treatment. The present results are similar to those of another study, suggesting that FAS treatment reverses cell apoptosis induced by PD98059 in ischemic preconditioning [38]. Based on current findings, activation of the MAPK signaling pathway is involved in FAS-induced BMSC migration.

For improvement of directed homing of BMSCs, many techniques have been developed, such as stem cell-based strategies or target tissue-based strategies [39]. Despite the promising literature, there are some limitations that make it difficult to generalize these results to the clinic. Compared with other strategies, FAS treatment, which itself was reported to promote axonal regeneration and enhance functional recovery after SCI, could exert positive effects on BMSCs [40]. In this study, we successfully labeled rat BMSCs with BrdU and found that FAS had no significant effect on the labeling rate of BrdU. The immunofluorescence assay of the spinal cord tissue showed that more FAS-pretreated BMSCs migrated to the injured area, which mainly distributed around the glial scar. This finding demonstrates that FAS could enhance the migration of BMSCs in vivo, which may help to establish scientifically verified strategies of cell transplantation therapy for SCI in the clinical situation. Although our study did not provide an in-depth assessment of histological and behavioral differences, overall, the FAS + BMSC group was superior in recovery of hindlimb motor function. In further studies, the specific mechanism of FAS in this combination therapy should be studied in more detail.

## 5. Conclusion

Taken together, we report for the first time that FAS promotes the migration of rat BMSCs in vitro, possibly by inducing actin stress fiber formation via activation of the MAPK signaling pathway. FAS also enhances the homing efficiency of BMSCs in a model of contusion SCI. Therefore, the combined strategy of BMSC transplantation and FAS might possess potential effect in stem cell therapy of CNS trauma.

## Data Availability

The data used to support the findings of this study are available from the corresponding author upon request.
